# Genetic Signature of Rapid IHHNV (Infectious Hypodermal and Hematopoietic Necrosis Virus) Expansion in Wild *Penaeus* Shrimp Populations

**DOI:** 10.1371/journal.pone.0011799

**Published:** 2010-07-26

**Authors:** Refugio Robles-Sikisaka, Andrew J. Bohonak, Leroy R. McClenaghan, Arun K. Dhar

**Affiliations:** Department of Biology, San Diego State University, San Diego, California, United States of America; University of Utah, United States of America

## Abstract

Infectious hypodermal and hematopoietic necrosis virus (IHHNV) is a widely distributed single-stranded DNA parvovirus that has been responsible for major losses in wild and farmed penaeid shrimp populations on the northwestern Pacific coast of Mexico since the early 1990's. IHHNV has been considered a slow-evolving, stable virus because shrimp populations in this region have recovered to pre-epizootic levels, and limited nucleotide variation has been found in a small number of IHHNV isolates studied from this region. To gain insight into IHHNV evolutionary and population dynamics, we analyzed IHHNV capsid protein gene sequences from 89 *Penaeus* shrimp, along with 14 previously published sequences. Using Bayesian coalescent approaches, we calculated a mean rate of nucleotide substitution for IHHNV that was unexpectedly high (1.39×10^−4^ substitutions/site/year) and comparable to that reported for RNA viruses. We found more genetic diversity than previously reported for IHHNV isolates and highly significant subdivision among the viral populations in Mexican waters. Past changes in effective number of infections that we infer from Bayesian skyline plots closely correspond to IHHNV epizootiological historical records. Given the high evolutionary rate and the observed regional isolation of IHHNV in shrimp populations in the Gulf of California, we suggest regular monitoring of wild and farmed shrimp and restriction of shrimp movement as preventative measures for future viral outbreaks.

## Introduction

Effective implementation of monitoring and control measures for viral epizootic outbreaks requires an understanding of the factors that underlie molecular evolution and population dynamics. This is also true for introduction of the infectious hypodermal and hematopoietic necrosis virus (IHHNV) into shrimp populations of the northern Pacific coast of Mexico. IHHNV is a single-stranded DNA-containing virus belonging to the family *Parvoviridae* [1]. The IHHNV genome is 4.1 kb long, with three open reading frames (ORFs) [2]. The left ORF encodes the non-structural proteins, the right ORF encodes the capsid protein, and the function of the middle ORF is unknown [2]. IHHNV is widely distributed in wild and farmed penaeid shrimp species in the Americas, Asia and Oceania [3], [4]. In the northwestern coast of Mexico, IHHNV affects two of the most economically important shrimp species, *Litopenaeus stylirostris* and *L. vannamei*. This viral pathogen is known to cause mortalities of up to 90% in juvenile and subadult individuals of *L. stylirostris* [5], and growth reduction and cuticular deformities in *L. vannamei* [6]. IHHNV was first detected in Hawaii in 1981 [7], and by 1987, its presence was confirmed in the southern region of the Gulf of California, Mexico [7]. In 1990, IHHNV caused major epizootics in farmed and wild *L. stylirostris* populations in the Gulf of California [8], [9]. The spread of the virus throughout the northwestern coast of Mexico occurred rapidly, so that IHHNV prevalence in wild shrimp stocks had reached almost 100% across the entire Gulf of California by 2005 [10]. Interestingly, high mortalities caused by IHHNV in farmed shrimp have not been recorded since the epizootics of 1990, and wild *L. stylirostris* fishery landings have increased since that time [9]. This has led to the presumption that *L. stylirostris* has developed resistance to IHHNV and/or the virus has reached an equilibrium with the host in terms of genes related to virulence [4], [9]. Although these hypotheses have yet to be formally tested, Tang and Lightner (2002) [4] cited limited nucleotide variation among IHHNV isolates from the Americas and Hawaii as support. The authors suggest that “the IHHNV genome has been stable” due to “the development of a more balanced host-pathogen relationship.” However, they did not quantitatively interpret current levels of genetic diversity in terms of the underlying evolutionary processes (mutation, genetic drift, gene flow and natural selection). Further, Tang and Lightner's [4] study was based on 14 isolates, of which only 4 were from Mexico.

To better understand the molecular evolution, population structure and dynamics of IHHNV in the Pacific coast of Mexico, we analyzed capsid protein gene sequences from wild shrimp populations in the region. With sample sizes much larger than in previous studies, we were able to make inferences about levels of variation, population structure and recent population history in this virus, leading us to question whether it should be regarded as a static evolutionary lineage.

## Materials and Methods

### Sample collection

**Figure 1:**
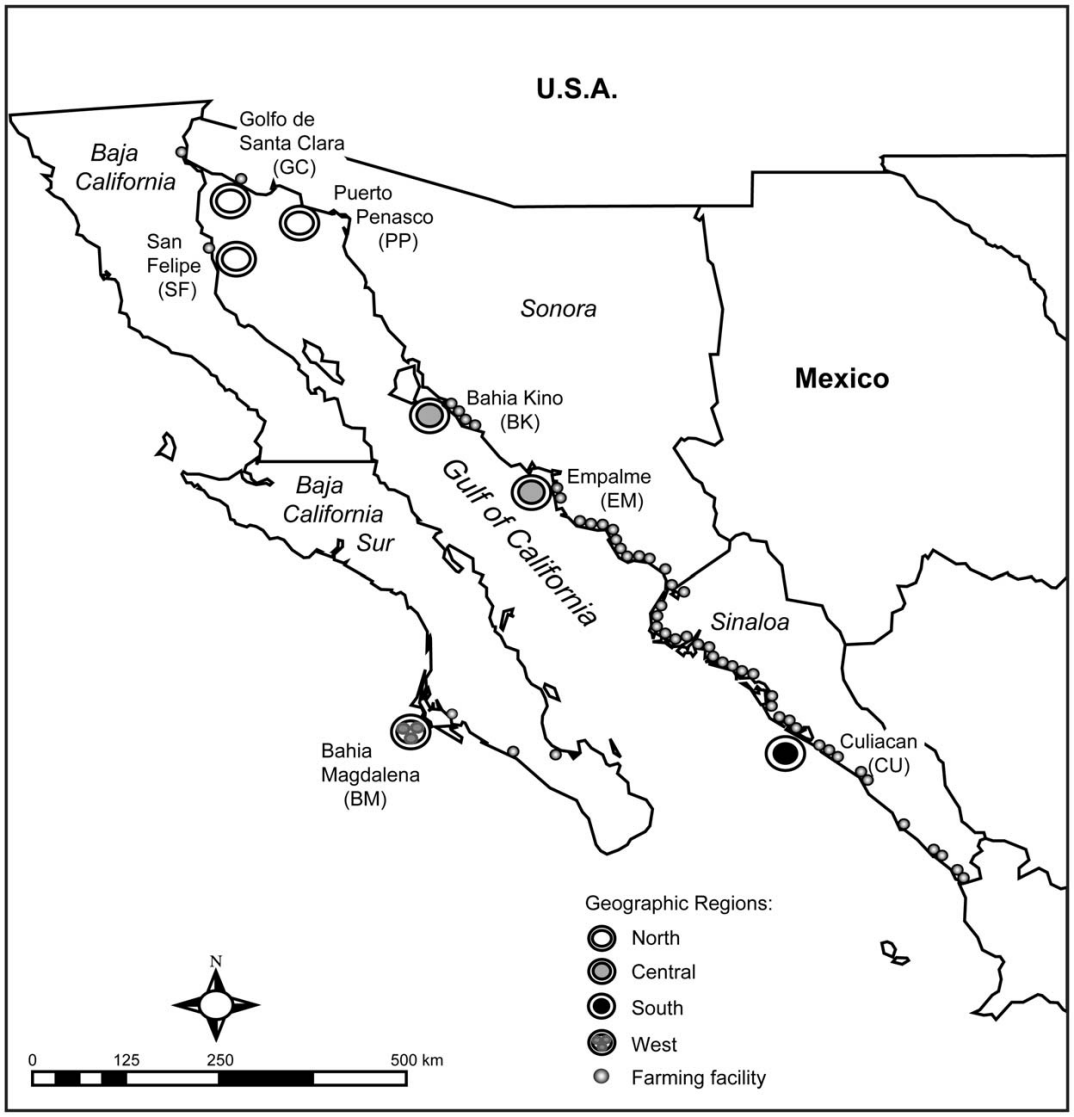
Sample collection sites and distribution of penaeid shrimp farms in Mexico during 2005. Concentric circles: collection sites; small circles: shrimp farming facilities. Number of shrimp farms located in Sinaloa n = 477, Sonora n = 119, Baja California n = 27 and Baja California Sur n = 8 [51].

Shrimp (*L. stylirostris*, *L. vannamei* and *Farfantepenaeus californiensis*) were collected from seven sites in the northwestern Pacific coast of Mexico from November 2004 to January 2005 (Figure 1; Table 1). Three sites were located in the northern region of the Gulf of California (San Felipe, Golfo de Santa Clara and Puerto Peñasco), two in the central region (Bahia Kino and Empalme), and one in the southern region (Culiacan). The final site, Bahia Magdalena, was located on the southwestern side of the Baja California Peninsula (Figure 1). After collection, shrimp were transported to the laboratory on dry ice, and stored at −80°C until further processing.

**Table 1:** Collection sites, species and number of individuals sequenced (n) and attempted for sequencing (N).

Collection site	N	n	Species
San Felipe, B.C. (SF)	16	13	*L. stylirostris*
Golfo de Santa Clara, Son. (GC)	15	15	*L. stylirostris*
Puerto Peñasco, Son. (PP)	14	8	*L. stylirostris*
Bahia Kino, Son. (BK)	11	10	*L. stylirostris* (8)
			*L. vannamei* (2)
Empalme, Son. (EM)	15	15	*L. stylirostris*
Culiacan, Sin. (CU)	16	14	*L.stylirostris* (10)
			*L. vannamei* (3)
			*F. californiensis* (1)
Bahia Magdalena, B.C.S. (BM)	16	14	*L. stylirostris*
Total	103	89	*L. stylirostris* (83)
			*L. vannamei* (5)
			*F. californiensis* (1)

### DNA isolation and sequencing of IHHNV capsid gene

Genomic DNA was extracted from 50–100 mg of shrimp tail muscle using DNAzol™ (Molecular Research Center Inc., Cincinnati, Ohio) following manufacturer's instructions. Extracted DNA was resuspended in 100 µl of tris EDTA buffer and stored at −20°C. The IHHNV capsid gene was amplified by PCR using primers CP1F (5′-GAT CAC CAG CAC GAC TTC CT -3′) and CP2R (5′- CGG GTA TAT ATT GCA CAT CGA A-3′) that were designed for this study based on a published IHHNV sequence (GenBank accession number AF273215) [2]. The reaction mixture for the PCR amplification contained 0.5 units of PCR Supermix High Fidelity or Platinum® PCR Supermix (Invitrogen, Carlsbad CA), 200 nM each of forward and reverse primers, 2.5 mM MgCl_2_, and 1µl of extracted genomic DNA (∼40 ng) in a 25 µl final reaction volume. Amplification parameters included an initial denaturation of 94°C for 2 min., followed by 35 cycles of 94°C for 30 sec, 55°C for 30 sec and 72°C for 1 min. A final extension of 72°C for 7 minutes was used. The amplified DNA was electrophoresed in a 1% agarose gel, and gel purified using a Qiaquick gel purification kit (Qiagen, Valencia, CA). Amplified DNA samples were directly sequenced in both directions using the CP1F and CP2R primers. Sequencing was performed at the High-Throughput Genomics Unit of the University of Washington (Seattle, WA).

### DNA sequence analysis

IHHNV nucleotide sequences were aligned using ClustalX [11]. The number of segregating (polymorphic) sites (S), number of synonymous and nonsynonymous substitutions, number of haplotypes (H), haplotype diversity (Hd), and nucleotide diversity (π, based on the Jukes-Cantor model of nucleotide substitution [12]) were calculated using DNAsp version 4.5 [13]. Nucleotide sequences of IHHNV capsid genes reported in this paper have been deposited to GenBank with the accession numbers GU906889 to GU906977 (Table S1).

### Population genetic analysis

Each collection site was operationally treated as a population (gene pool) for the initial analyses. Arlequin v.3.0b [14] was used to determine statistically significant associations between pairs of populations based on their haplotype distributions. The null hypothesis of random haplotype distributions across populations was tested using 100,000 Markov chain steps, 10% of which were discarded as burnin. Genetic differences among pairs of populations were quantified using Φ*_ST_* [15] (10,000 permutations). This statistic is analogous to the commonly used *F_ST_*, except that Φ*_ST_* considers the genealogical relationships among alleles as well as their frequencies in each population. The uncorrected distance between pairs of haplotypes was used for these calculations. Significant association between genetic and geographic distances among pairs of populations was assessed using Mantel tests as implemented in the Isolation by Distance Web Service [16] (30,000 randomizations).

### Phylogenetic analysis

To determine evolutionary relationships among the Mexican IHHNV isolates obtained in this study and compare them to other geographic isolates, 14 IHHNV capsid protein sequences isolated from *L. stylirostris* and *P. monodon* in different geographic locations were downloaded from GenBank and included in phylogenetic analyses (Table S1). Eight *P. monodon* IHHNV sequences from GenBank were used for rooting the Bayesian tree (accession numbers AY124937, DQ228358, AY102034, AY355307, AY362547, EU675312, GQ411199, FJ169961). We first constructed a haplotype network using statistical parsimony in the program TCS v1.21 [17]. This algorithm takes into account haplotype frequencies, and accounts for the persistence of ancestral haplotypes in contemporary gene pools [18]. For comparison, a Bayesian inference phylogenetic analysis was performed in Mr. Bayes [19]. Bayesian inference has several advantages over other phylogenetic methods, including complex models of sequence evolution and the use of prior information [20].

To perform the Bayesian phylogenetic analysis, the data set of 61 unique IHHNV capsid sequences (47 unique haplotypes obtained in this study and 14 sequences from GenBank) was aligned using ClustalX [11]. The likelihood settings from the best-fit model of nucleotide substitution for the aligned data set (TVM+I+G) were selected based on the Akaike Information Criterion (AIC) using the computer program jModeltest [21]. Markov chain Monte Carlo (MCMC) parameters included a run time of 3×10^6^ generations, and sampling every 1000 generations. Convergence was acknowledged when the standard deviation of the split frequencies dropped below 0.01; parameter files from the Bayesian analysis were analyzed in TRACER [22] to verify chain convergence and recognized when the estimated sample size (ESS) exceeded 200. The 50% majority rule consensus tree was visualized using FigTree v1.1.2 [23]. Clade support was estimated by Bayesian posterior probabilities.

To assess the influence of multiple convergent mutations (i.e., molecular homoplasy) on the inferred IHHNV phylogenetic tree, the data set used in the Bayesian analysis was analyzed to calculate the number of parsimony informative characters with homoplasy using the computer program MacClade v.4.08 [24].

### Geographic origin of IHHNV within the northwestern coast of Mexico

Lightner and colleagues documented the initial history of IHHNV outbreaks in Mexico and noted that the virus was first detected in the southern region of the Gulf of California [7]. However, the area of initial detection does not necessarily represent the area of introduction. To make inferences about the geographic location of ancestral IHHNV lineages in this region, we took two approaches: statistical parsimony and ancestral DNA character state reconstruction. In statistical parsimony, the most frequent and most connected haplotype is regarded as being ancestral [18]. The analysis of ancestral IHHNV lineages using statistical parsimony was performed using TCS v1.21 [17] as described above. Ancestral DNA character state reconstruction was performed under a stochastic model of character state change in the computer program SIMMAP [25]. The program elements included an IHHNV topology estimated from the MrBayes tree file (excluding 10% burnin) and a GTR+I+G model of nucleotide substitution as determined by Modeltest v.3.7 [26]. Ancestral DNA character states were inferred for the most basal tree node using one Asian isolate (accession number AY102034) as an outgroup. IHHNV ancestral DNA character states were compared to consensus sequences from the different regions in the Gulf of California, and the number of DNA state similarities was recorded. The consensus sequence with the highest number of similarities with the inferred ancestral DNA characters was considered most likely to be ancestor to the IHHNV lineages sampled for this study.

### Estimating the rate of nucleotide substitution and inference of demographic history

The rate of nucleotide substitution and historical effective number of infections were estimated using the computer program BEAST [27]. BEAST uses a Bayesian MCMC approach to evaluate evolutionary hypotheses without conditioning to a tree topology, and it has been used to estimate the rate of evolution, divergence times and temporal changes in effective population size for a wide array of taxa [e.g.], [ 28]–[32]. The rate of nucleotide substitution per site per year was estimated from an independent data set that included a total of 18 dated IHHNV capsid DNA sequences. Fourteen of these sequences are globally distributed isolates taken from GenBank and four were sequenced from the Gulf of California for this study (Table S1). Each of the 18 IHHNV capsid sequences was nearly 1 kb in length (969 bp in all cases but one of 870 bp). The sampling time interval of these sequences is 21 years (isolation years ranging from 1986 to 2007). The sequence from 1986 predates the extensive epizootics in the Gulf of California in 1990, and is close to the first detection of IHHNV in 1981 [7]. Evolutionary model parameters incorporated in BEAST included a SDR06 model of nucleotide substitution, which uses a HKY substitution model with a Gamma site heterogeneity and two codon partitions (one for the first and second codon positions and a second for the third). The rate of nucleotide substitution for this data set was estimated under both strict and relaxed (uncorrelated lognormal) molecular clocks and five demographic models (constant population size, exponential growth, expansion growth, logistic growth and Bayesian skyline plot: BSP). The best-fit model for the calculation of the IHHNV nucleotide rate was determined by comparing the marginal likelihood estimates of each model [33] using Bayes factors as implemented in TRACER [22]. Each MCMC chain was run for 10–20×10^6^ generations, with 10% eliminated as burnin, which seemed appropriate based on the visual inspection of the run trace, and effective sample sizes were >200.

Historical changes in effective population size *N_e_* can be inferred from the BSP [34], [35]; because IHHNV is a virus, we interpreted this parameter as the effective number of infections in units of generations/year. Given that the IHHNV generation time is unknown, we did not attempt to translate this parameter into the actual number of infections. The BSP derives from a coalescent process used to estimate the effective number of infections through time from a sample of gene sequences. Since the IHHNV capsid gene copies sequenced in this study were obtained from shrimp collected only one year apart and can be considered contemporaneous, time calibration was incorporated by fixing the nucleotide rate of substitution/site/year based on the dated IHHNV sequence data set (Table S1). To determine the sensitivity of inferred population dynamics to mutation rate, two additional BSPs were constructed using the 25th and 75th percentile of the highest posterior density (HPD) interval corresponding to the mean mutation rate estimate. To maintain the independence of both IHHNV data sets (dated and contemporaneous), four sequences that were used to determine the rate of nucleotide substitution were removed from this analysis (accession numbers: GU906908, GU906919, GU906937, GU906953). Thus, a total of 85 IHHNV sequences was used in the BSP analysis. Additional parameters in the analysis included a SDR06 model of nucleotide substitution, a strict molecular clock, a constant skyline model with 17 size groups in the prior, and a uniform prior population size with an upper bound of 1×10^7^. The MCMC algorithm was run for 30×10^6^ generations, with sampling every 3000 generations and 10% burnin. Visual examination of parameter and likelihood plots in TRACER v.1.4 [22] confirmed that this burnin was sufficient.

To determine whether inferences from the Bayesian skyline plot might be biased by IHHNV population subdivision, we also performed separate BSP analyses for each region (northern: San Felipe, Golfo de Santa Clara and Puerto Peñasco; central: Bahia Kino and Empalme; south: Culiacan). Bahia Magdalena (BM) was not included in the analysis due to its geographical separation from the Gulf of California. The constant skyline model included 4–8 size groups depending on the data set size, and the MCMC algorithm was run for 10×10^6^ generations, with sampling every 1000 generations. The remainder of the parameters were the same as described above.

### Recombination and selection analysis

The 89 IHHNV capsid sequence alignment was analyzed for evidence of recombination breakpoints using the Genetic Algorithms for Recombination Detection (GARD) tool [36]. Suggestions of positive, or diversifying, selection acting in IHHNV codon sites were evaluated using three different codon-based maximum likelihood methods: Random Effects Likelihood (REL), Fixed Effects Likelihood (FEL) and Single Likelihood Ancestor Counting (SLAC) [37]. These methods detect site-specific signatures of positive selection by estimating the ratio of nonsynonymous/synonymous substitutions (dN/dS) at every codon. The dN/dS ratio has been widely used as an indicator of natural selection, with ratio values less than 1 consistent with negative or purifying selection acting on amino acid-altering mutations, and values greater than 1 suggesting positive selection to increase the number of replacements. Briefly, the REL method estimates dN and dS values from an independent distribution of rates chosen *a priori*. In contrast, the FEL method estimates dN and dS directly from the data at each codon site. The SLAC method calculates these parameters from reconstructed ancestral sequences and compares observed dN and dS against the expected corresponding values. For all codon-based analyses, the 89 IHHNV sequence alignment was in-frame, with the first nucleotide corresponding to the first codon position.

We also inferred whether positive selection has acted in specific IHHNV lineages, leading to differential selective pressure across the IHHNV phylogeny. This was performed using the Genetic Algorithm (GABranch) method [38], which tests models with variable rates of dN/dS and allows each branch of a phylogeny to select its best-fit evolutionary rate using the small sample AIC score.

## Results

### Amplification of IHHNV capsid protein gene

A 1048 bp amplicon representing the IHHNV capsid protein was amplified using the primers CP1F and CP2R from 89 shrimp across the seven collection sites (Table 1). After trimming incomplete ends that were unavailable for some isolates, a standardized length of 969 bp was used for analysis. The IHHNV capsid gene could not be amplified in samples collected from an eighth site in Mazatlan, located in the southern part of the Gulf of California. A real-time PCR study of the same shrimp samples [10] determined that the IHHNV load was lowest in the Mazatlan samples, likely contributing to the failure of these samples to amplify. The majority of the IHHNV sequences were amplified from *L. stylirostris* (n = 83) followed by *L. vannamei* (n = 5) and one from *F. californiensis* (Table 1).

### Population genetic analysis

The 969 bp multiple alignment of the 89 IHHNV capsid gene sequences contained 62 polymorphic sites (6.3%) and 64 mutations (two parsimony informative sites, 51 and 310, had three variants). Of the 64 substitutions, 28 corresponded to synonymous and 36 to nonsynonymous substitutions (Table 2). No insertions or deletions were observed. The highest level of nucleotide diversity was found between nucleotide positions 671 and 730 (π = 0.0193).

**Table 2:** Genetic diversity in the 969 bp capsid protein region of IHHNV isolated from penaeid shrimp in the northern Pacific coast of Mexico.

		Nucleotide Diversity				Haplotype Diversity	
Geographic Location	n	S	SynonymousSubstitutions	NonsynonymousSubstitutions	π	H	Hd
Golfo de Santa Clara B.C. (GC)	15	13	9	4	0.0022	7	0.79
Puerto Peñasco, Son. (PP)	8	3	1	2	0.0007	2	0.25
San Felipe, Son. (SF)	13	4	1	3	0.0009	4	0.65
Bahia Kino, Son. (BK)	10	22	8	14	0.0089	8	0.95
Empalme, Son. (EM)	15	11	3	8	0.0026	7	0.88
Culiacan, Sin. (CU)	14	20	6	14	0.0084	11	0.95
Bahia Magdalena, B.C.S. (BM)	14	15	5	10	0.0059	8	0.89
Overall	89	62	28	36	0.0065	47	0.96

There were 47 unique haplotypes, with only 2 shared among sites. One haplotype was present in seven individuals each from San Felipe and Golfo de Santa Clara, and a second was found in two shrimp from Empalme and four from Bahia Kino.

Average nucleotide and haplotype diversity estimates were lower in the northern localities (π: 0.00178; Hd: 0.811) than in the central (π: 0.00614; Hd: 0.927) and southern regions (π: 0.00849; Hd: 0.956). Both measures of genetic diversity decreased with increasing latitude (Spearman rank correlations, one-tailed test, p<0.05 for each; Table 2). The overall amino acid diversity was almost twice as high as the nucleotide diversity value, reflecting the higher number of replacement than silent substitutions (Table 2). Genetic divergence among the seven sampling sites was moderate, but highly significant (AMOVA: Φ*_ST_* = 0.369, p<0.0001). Exact tests of haplotype distributions showed significant differentiation between all pairs of sites (p values from 0.0001 to 0.03), except San Felipe vs. Golfo de Santa Clara. Pairwise Φ*_ST_* values also showed that all seven populations were genetically different from each other (p<0.05; Table 3). However, genetic differentiation was unrelated to geographic distance (Mantel Test for isolation by distance, p>0.05).

**Table 3:** Pairwise Φ*_ST_* among seven populations of IHHNV isolated from penaeid shrimp in the northern Pacific coast of Mexico.

	Golfo de	Puerto				
	Santa Clara	Peñasco	San Felipe	Bahia Kino	Empalme	Culiacan
Puerto Peñasco	0.31**	-				
San Felipe	0.06*	0.48**	-			
Bahia Kino	0.25**	0.28**	0.27**	-		
Empalme	0.54**	0.63**	0.60**	0.27**	-	
Culiacan	0.43**	0.43**	0.45**	0.21*	0.43**	-
Bahia Magdalena	0.37**	0.42**	0.40**	0.13*	0.31**	0.28**

(*) significant at p<0.05; (**) significant after Bonferroni correction for 21 tests (p≤0.002).

### Phylogenetic analysis of IHHNV isolates

The data set of 61 unique sequences used for the phylogenetic analysis contained 231 polymorphic sites (24%), of which 194 were parsimony informative. Although this data set contained 13 distantly related sequences from outside of the study area, only 48 sites (5%) showed any degree of homoplasy (i.e., convergent mutations) on the final Bayesian tree, and only 17 sites were inferred to have 3 or more mutations.

**Figure 2:**
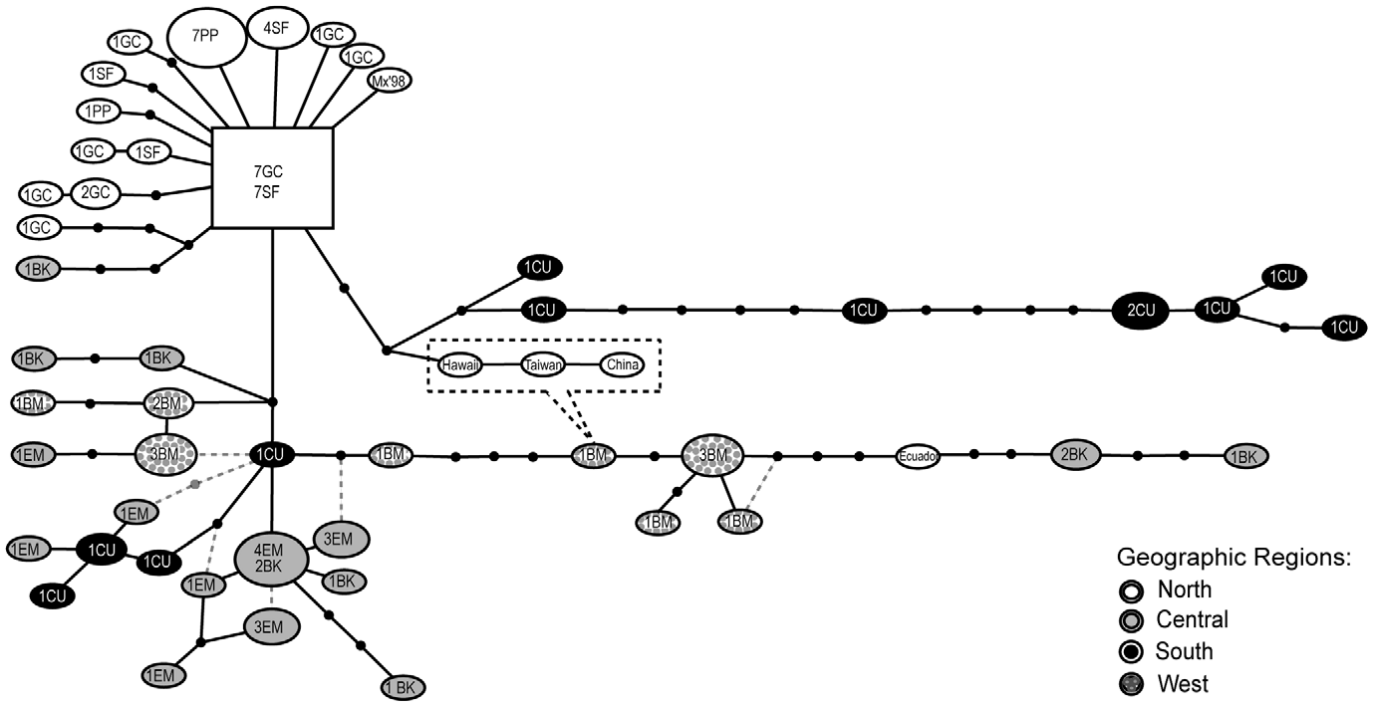
Haplotype network of IHHNV genotypes. Population codes: San Felipe (SF), Golfo de California (GC), Puerto Peñasco (PP), Bahia Kino (BK), Empalme (EM), Culiacan (CU) and Bahia Magdalena (BM). Circle color corresponds to the region of haplotype origin: white: north, gray: central, black: south, dotted: west coast of the Baja California Peninsula. Size is proportional to the number of individuals in the corresponding haplotype. The ancestral haplotype is represented by the rectangle, small black circles are unsampled haplotypes, and each line represents one mutational step. Dashed lines are haplotype relationships statistically undefined by the TCS algorithm. The dashed box reflects the connection of the Asian haplotypes to BM and BK haplotypes in the Bayesian phylogenetic tree.

The IHHNV haplotype network produced four distinct clades (Figure 2). The clade formed by the northern samples (upper left panel in Figure 2) included all haplotypes from San Felipe, Golfo de California and Puerto Peñasco in addition to one haplotype from the central site of Bahia Kino. This clade is characterized by close relationships among the 14 haplotypes (maximum divergence of 4 mutational steps). The central clade (lower left panel in Figure 2) is characterized by a mixture of haplotypes from the central, southern and western regions, with some relationships statistically undefined (e.g., due to reticulations or convergence). The southern and western clades were more divergent, with maxima of 15 and 12 mutational steps within each clade respectively. The southern clade included haplotypes exclusively from Culiacan, whereas the western clade included both western and central haplotypes.

**Figure 3:**
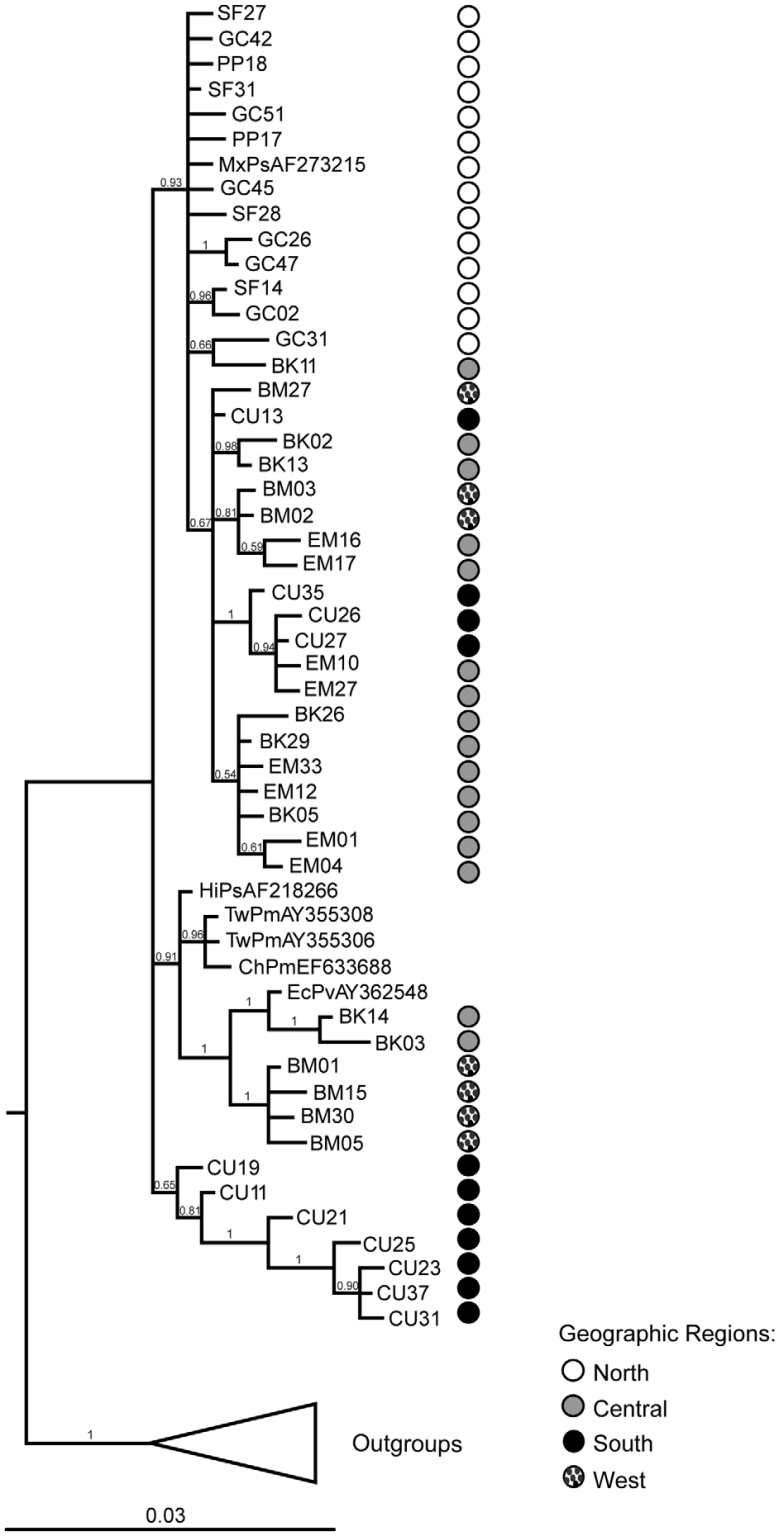
Bayesian phylogenetic tree of IHHNV isolates. Posterior probabilities are indicated in the branch nodes. The tree was rooted using Asian isolates of IHHNV infecting *P. monodon* as outgroups and collapsed for clarity. Circles represent the region of haplotype origin in the northwestern coast of Mexico; white: north; gray: central; black: south; dotted: west coast of the Baja California Peninsula.

A 50% majority-rule tree obtained from the Bayesian analysis is shown in Figure 3. All Mexican isolates formed a highly supported monophyletic group. Similar to the haplotype network, the Bayesian tree contained a monophyletic clade with only southern haplotypes, and a second monophyletic clade that showed close relationships among western, central and Asian haplotypes. However, the majority of haplotypes fell into a single large clade with representatives from all areas in a partially unresolved polytomy. Some of the general structure of the haplotype network is also evident in this mixed clade, such as the short mutational distances among northern haplotypes, and affinities between some central haplotypes and some southern and western haplotypes. Overall, the signal of IHHNV geographic structure was observed in subsets of the southern and western haplotypes in clades with high posterior probabilities (Figure 3). However, posterior probabilities for the most basal nodes (closer to the root) were generally low.

### Geographic origin of the ancestral IHHNV lineage

Based on statistical parsimony assumptions of haplotype frequency and connectedness, a northern haplotype was inferred to be the ancestor of the sampled IHHNV lineages, since it is present in 14 individuals and is only 1 to 2 mutational steps away from 10 other haplotypes in the region (Figure 2). The IHHNV consensus sequences from the north and southern regions differed in nine nucleotide sites that provided the basis for ancestral DNA state reconstructions. Eight of the nine inferred ancestral DNA characters matched the northern consensus sequence, and only one corresponded to the southern consensus sequence (Table 4).

**Table 4:** Comparison of nucleotide site differences in the north and south consensus sequences, and the corresponding ancestral character state posterior probabilities.

Site	North	South	
number	consensus site	consensus site	Ancestral DNA character
19	A	G	A (89%) G (10%)
51	A	G	A (99%) G (1%)
363	G	A	G (21%) A (78%)
453	C	T	C (62%) T (35%)
661	G	A	G (80%) A (19%)
662	G	A	G (80%) A (19%)
663	C	G	C (90%) G (4%)
683	G	C	G (89%) C (3%)
718	A	C	A (82%) C (17%)

### Rate of nucleotide substitution and population demography of IHHNV

The mean rate of nucleotide substitution estimated from dated IHHNV capsid sequences under assumptions of both strict and relaxed molecular clocks were comparable and unexpectedly high for a DNA virus such as IHHNV. The relaxed molecular clock was favored over the strict clock, as the 95% highest posterior density interval (HPD) of the standard deviation of the lognormal marginal posterior distribution excluded zero (mean 0.764; 95% HPD 0.232–1.383). The relaxed molecular clock model also had a larger marginal likelihood according to the Bayes factors comparison (−2696.62 for the strict molecular clock compared to −2690.78 for the relaxed molecular clock). Mean mutation rates obtained with the five demographic models under the relaxed molecular clock ranged from 1.39 to 4.89×10^−4^ substitutions/site/year. Comparison of the marginal likelihoods calculated by Bayes factors showed that the expansion growth model had the largest marginal likelihood value, and is therefore the best-fit population growth model for IHHNV. The expansion growth model had a mean mutation rate of 1.39×10^−4^ (95% HPD 3.34×10^−5^–2.91×10^−4^). The rate of substitution under strict molecular clock assumptions and constant population size was very similar, with a mean mutation rate of 1.81×10^−4^ (95% HPD 2.61×10^−5^–3.39×10^−4^).

**Figure 4:**
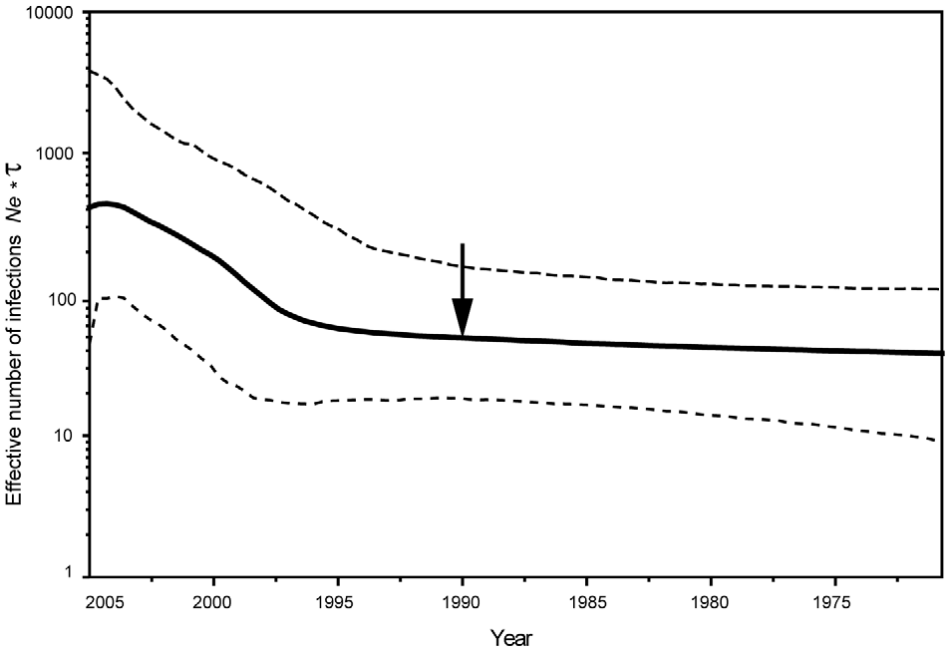
Bayesian skyline plot. The Y axis represents the effective number of infections, Ne*τ, where *Ne* is the effective number of infections and τ is the generation time. The black line represents the mean estimate of the effective number of IHHNV infections through time, and the dashed lines represent the 95% HPD. The arrow indicates 1990, the year of IHHNV epizootics in the Gulf of California.

Historical IHHNV population demography in the northwestern Pacific coast of Mexico was inferred from a Bayesian skyline plot incorporating the mean rate of 1.39×10^−4^ substitution/site/year for the 85 sequences obtained in this study (excluding the 4 used in the molecular clock estimate). The analysis suggested that IHHNV may have been present in Mexican penaeid shrimp population in the early 1970's, has been slowly increasing since the mid 1980's, and began an exponential phase of growth in approximately the early 1990's (Figure 4). These dates correspond very closely to historical records of IHHNV in the Gulf of California, with the virus first detected in wild shrimp populations from the region in 1987 [7], and the 1990's epizootics in cultured and wild shrimp populations [8], [9]. The most recent portion of the plot suggests that growth in the IHHNV population may have slowed or even leveled off, although the 95% HPD is too wide to make any definitive conclusions. The population increase that begins in the 1990's was also visible in the BSPs that used the 25% (8.33×10^−5^) and 75% (2.23×10^−4^) estimated mutation rates (data not shown). However, the timing of the population expansion differed by approximately 10 years (the late 1990's for the 75% rate, and the late 1980's for the 25% rate). Our analysis suggests that the common ancestor of the sampled IHHNV isolates dates to the early 1970's, which coincides with the assumed time of IHHNV introduction into the region [39]. Despite the relatively large confidence intervals, the BSP suggests that by 2005, the IHHNV effective number of infections had increased substantially over the previous three decades. Gradual long-term population growth was also suggested when the IHHNV data set was subdivided by geographic region (northern, central and southern; data not shown). The very recent population increase present in Figure 4 was also pronounced in the central region, although less dramatic in the northern and southern regions.

No evidence of recombination was found at any codon site in the IHHNV nucleotide alignment. Signals of natural selection were found in some codons, the number of which varied depending on the genetic algorithm used. The overall mean dN/dS ratio of the IHHNV capsid region analyzed as estimated by the SLAC method was 0.493. Only codon 7 was found to be positively selected under the three methods (significance threshold of p = 0.1 for SLAC and FEL algorithms; Bayes factor = 50 for REL). The REL method found five additional positively selected codons (21, 104, 136, 221, 249). Negatively selected codons under the three algorithms included codons 66, 151 and 213. Both FEL and REL algorithms detected additional negatively selected codons (17, 50, 66, 81, 134, 151, 172, 208, 209, 213, 272, 275). Other negatively selected codons detected by the REL method were 80, 91, 92, 118, 121,163, 180, 227, 233, 249, 262.

The GABranch analysis suggested surprisingly high levels of positive selection in 13 lineages of the IHHNV genealogy. Specifically, three lineages found in the northern region, six in the central and four in the south showed the highest possible dN/dS values (i.e., only nonsynonymous substitutions in those lineages), supported by at least 98% of the models tested.

## Discussion

### Genetic diversity of IHHNV

The history of IHHNV in the northwestern Pacific coast of Mexico provides a unique opportunity to study the molecular evolution and population dynamics of a marine parvovirus after its introduction into a new environment. We found that IHHNV haplotype and nucleotide diversity is higher than previously reported. Tang and colleagues analyzed the capsid gene of 14 IHHNV isolates from the Americas that included only 4 samples from the Gulf of California, and reported 1.3% segregating sites in the capsid protein gene [39]. Based on this observation, the authors suggested that the IHHNV genome was stable or had reached an equilibrium with the host. However, we found that IHHNV in the Gulf of California is almost five times more variable than Tang and colleagues estimated. We cannot easily attribute this to technical issues such as polymerase errors, since we used high fidelity enzymes and sequenced amplicons in both directions. Rather, the higher estimates of genetic variation found in this study represent a larger IHHNV sample size, collected from multiple genetically distinct populations in the region.

We found that genetic diversity is not uniform across geographic regions in the northwestern coast of Mexico. In fact, IHHNV localities in the central and southern regions of the Gulf of California are an order of magnitude more variable than those in the northern region. The statistical parsimony network and Bayesian phylogenetic tree also show more geographic structure in the southern and western regions, with the central region representing an admixture of isolates of different geographic origins. These differences may be explained in light of the marine and biogeographic features present in the Gulf of California. The northern area of the Gulf is characterized by a unique cyclic current system that limits interactions with the central and southern regions of the Gulf [40]. Regional differentiation is also promoted by geographic barriers; the islands Isla Tiburon in the eastern coast and Isla Angel de la Guarda in the western coast (Figure 1) partially block the northern and central portions of the Gulf. These features may limit gene flow between regions. However, it should be noted that not all gene flow is obstructed, as suggested by a Bahia Kino haplotype closely related to those found in the northern clade.

The mixture of haplotypes evident in the haplotype network and Bayesian analysis suggests high genetic exchange among the central and southern regions and across the Baja California Peninsula. Interestingly, Bahia Kino and Bahia Magdalena haplotypes are closely related despite being separated by the strip of land that represents Baja California Sur. Natural movement of aquatic organisms across the coasts of the Baja California Peninsula is highly unlikely due to the large geographic distance. However, association of geographically distant haplotypes could be due to human-mediated transport of IHHNV infected broodstock and post-larvae between these two regions. In shrimp aquaculture, a common practice is the capture of wild shrimp in different life stages for seed, spawners and broodstock [41] and their transport to aquaculture facilities. It is possible that shrimp growers may have obtained IHHNV-infected larvae from a common supplier and the virus was inadvertently spread to geographically distant coastal farms.

### IHHNV introduction to northwestern coast of Mexico

The statistical parsimony haplotype network provided a clear indication that the geographic location of IHHNV introduction is in the northwestern coast of Mexico. The rooting algorithm used by statistical parsimony assumes that gene pools contain multiple, identical sets of haplotypes that mutate periodically producing a mixture of coexisting old and new haplotypes [18]. Under this assumption, the ancestral haplotype is the most abundant and is highly connected to descendant haplotypes (Figure 2). We also note that reconstruction of ancestral DNA character states showed that 8 of 9 inferred ancestral states for sites that differ between northern and southern consensus sequences correspond to nucleotides present in the northern consensus sequence (Table 4). Our conclusion that IHHNV originated in the northern region of the Gulf of California is consistent with the development of intensive penaeid shrimp aquaculture methods in the northern Gulf of California in 1973 [7]. We hypothesize that the virus spread at some point throughout the rest of the Gulf, until it was detected in 1987 in the southern region.

### Nucleotide substitution rate in IHHNV and population demography

We estimated a rate of IHHNV nucleotide substitution of 1.39×10^−4^ substitution/ site/ year, which is unexpectedly high for a single-stranded DNA virus, and comparable to RNA viruses which evolve at a rate of 10^−3^ to 10^−5^ substitutions/site/year [42], [43]. The 95% HPD for the IHHNV rate of evolution (3.34×10^−5^–2.91×10^−4^) also excludes slow, large DNA virus-like rates of evolution. It is generally assumed that DNA viruses evolve at a similar rate to that of their hosts due to viral dependence on the host's cellular machinery for replication [44], [45]. Although these estimates could be influenced by the use of only 18 sequences in the analysis, we believe that this data set provides a good approximation of the actual rate of nucleotide substitution due to the length of the sequences, range of sampling dates and genetic diversity observed (overall mean of 0.34 substitutions per site for this data set). We also note that a growing number of parvoviruses infecting mammals and begomoviruses infecting plants have been shown in recent years to have similar substitution rates [46], [45]. These include canine parvovirus (CPV) (1.7×10^−4^), feline panleukopenia parvovirus (FPLV) (9.4×10^−5^) [46], human B19 erythrovirus (1×10^−4^) [45], human parvovirus B19 [47], porcine circovirus 2 [48] and the ssDNA containing begomovirus, tomato yellow leaf curl virus (TYLCV, 2.88×10^−4^) [31]. IHHNV is the first marine invertebrate parvovirus for which such high rates of nucleotide substitution have been reported.

Additional support to our estimated substitution rate comes from close correspondence between IHHNV population growth inferred by the Bayesian skyline plot and the time of first detection and viral epizootics in the northwestern coast of Mexico. It is possible that the BSP was influenced by selection in some of the lineages and population subdivision (although the region-specific BSPs showed the same qualitative patterns). However, both the mean and the 25% estimated rates of nucleotide substitution produced population dynamics that fit reasonably well with the IHHNV epizootic history in the Gulf of California, although the 75% estimated rate suggests a younger population history inconsistent with the historical records. The BSP traces the IHHNV most recent common ancestor back to the early 1970's, and the introduction of *Penaeus monodon* for aquaculture at that time has previously been suggested as the source of IHHNV in Mexico [39]. The effective number of infections slowly increased until the beginning of the 1990's, when it began to resemble exponential growth (Figure 4). If the founding IHHNV isolates were immigrant haplotypes from an older IHHNV population, possibly from Asia [49], then the Mexican IHHNV tree genealogy would coalesce prior to the actual introduction of the pathogen into the region. Finally, the fact that IHHNV prevalence had reached almost 100% in this region by 2005 [10] seems to be reflected in the most recent portions of the BSP (Figure 4).

The high levels of diversity we found in IHHNV contrast with previous suggestions of IHHNV low variation and stability [4], [8], [9], [39]. Interestingly, we found no evidence of recombination in our data set even though recombination is considered to be an important source of genetic variation in parvoviruses [50]. Although the mean dN/dS ratio obtained for the IHHNV capsid is consistent with negative or purifying selection, this average value inadequately reflects the diversity of evolutionary forces that may be acting. Codon-by codon analyses showed that multiple IHHNV codons and lineages were under positive selection for accelerated rates of nucleotide substitution, relative to neutral expectations for drift and mutation alone. Positively selected branches were found in all localities except in the Gulf of Santa Clara. Overall, three lineages from the northern, six lineages from the central and four lineages from the southern regions showed particularly high levels of dN/dS. Interestingly, the highest levels of IHHNV genetic diversity were also found in the central and southern regions. These regions are currently the most important producers of cultured shrimp in Mexico, with 477 shrimp farms in Sinaloa (southern region) and 119 in Sonora (central region) alone [51]. Since there are many contact opportunities between wild and the farmed populations of shrimp, these high levels of aquaculture presumably inflate population sizes of both host and pathogen. In conjunction with the high evolutionary rate of IHHNV, these population sizes contribute to increased genetic diversity. Thus, we interpret reduction in virulence across the Gulf of California primarily in terms of pathogen evolution. Specifically, we hypothesize an inherent tradeoff between transmission rate and virulence that has changed over time as IHHNV evolved. Future research that elucidates the basis of this tradeoff in IHHNV would be particularly informative.

IHHNV is a dynamic entity, with genetic differentiation among geographic regions in the Gulf of California. The presence of several quickly evolving lineages in the central and southern regions of the Gulf of California might be viewed as geographic “hotspots” of IHHNV variability that could lead to the emergence of virulent strain(s) following changes in the host or environmental conditions. The potential for an economically devastating IHHNV outbreak is further enhanced by current global aquaculture practices, with the spread of viral diseases in shrimp aquaculture attributable to the movement of infected animals among countries and even continents [49]. Because shrimp farms are generally located in coastal regions that also provide a natural habitat for wild shrimp, there are ample opportunities for admixture of wild and captive shrimp populations. As a result, a lethal strain of IHHNV originating in the wild can potentially be introduced into cultured populations, and vice versa. Routine genetic monitoring of IHHNV in wild and cultured shrimp populations might aid management efforts in this regard. In 1999–2000, a new strain of TSV (Taura syndrome virus, one of the most important RNA viral pathogens of penaeid shrimp worldwide) caused significant economic losses in Mexican shrimp farms. Initially, the new strain (TSV Mx2000) escaped detection since it did not react to the monoclonal antibody used for the routine detection of TSV by immunohistochemistry (ISH). Subsequent cloning and sequencing revealed that the new strain contained unique capsid gene mutations that contributed to detection failure in the ISH method [52]. Genetic screens for IHHNV may allow the identification of otherwise undetectable strains, as well as shed light on the unknown determinants of changes in virulence.

IHHNV dynamics in the Gulf of California should, therefore, warrant frequent genetic monitoring in both wild and farmed shrimp populations, as well as stricter enforcement of biosafety measures. Because our data show evidence for regional isolation of IHHNV in shrimp populations, restriction of shrimp movement is likely to be an effective management tool in limiting spread of the virus. The assumption that IHHNV is a stable, static virus understates the potential for a new virulent strain to arise, leading to epizootics similar to those observed in the early 1990's.

## Supporting Information

Table S1 Details and GenBank accession numbers of the IHHNV capsid sequences used in this study.
(0.11 MB DOC)
