# Supplementary material for: Genetic Signature of Rapid IHHNV (Infectious Hypodermal and Hematopoietic Necrosis Virus) Expansion in Wild Penaeus Shrimp Populations
Source: PLoS One. 2010 Jul 26;5(7):e11799. doi: 10.1371/journal.pone.0011799 (PMC2909959; doi:10.1371/journal.pone.0011799)
Supplement: Table S1 — Details and GenBank accession numbers of the IHHNV capsid sequences used in this study. (0.11 MB DOC) [file pone.0011799.s001.doc]

| **Isolate Name** | **Host Species** | **Isolation Year** | **Geographic Location** | **Accession Number** |
| --- | --- | --- | --- | --- |
| SF 02 | *L. stylirostris* | 2004 | Mexico | GU906897 |
| SF 06 | *L. stylirostris* | 2004 | Mexico | GU906901 |
| SF 10 | *L. stylirostris* | 2004 | Mexico | GU906891 |
| SF 12 | *L. stylirostris* | 2004 | Mexico | GU906893 |
| SF 13 | *L. stylirostris* | 2004 | Mexico | GU906894 |
| SF 14 | *L. stylirostris* | 2004 | Mexico | GU906895 |
| SF 15 | *L. stylirostris* | 2004 | Mexico | GU906896 |
| SF 17 | *L. stylirostris* | 2004 | Mexico | GU906900 |
| SF 27 | *L. stylirostris* | 2004 | Mexico | GU906890 |
| SF 28 | *L. stylirostris* | 2004 | Mexico | GU906892 |
| SF 30 | *L. stylirostris* | 2004 | Mexico | GU906898 |
| SF 34 | *L. stylirostris* | 2004 | Mexico | GU906899 |
| SF 31 | *L. stylirostris* | 2004 | Mexico | GU906889 |
| GC 02 | *L. stylirostris* | 2004 | Mexico | GU906904 |
| GC 14 | *L. stylirostris* | 2004 | Mexico | GU906905 |
| GC 15 | *L. stylirostris* | 2004 | Mexico | GU906906 |
| GC 24 | *L. stylirostris* | 2004 | Mexico | GU906907 |
| GC 26 | *L. stylirostris* | 2004 | Mexico | GU906908 |
| GC 28 | *L. stylirostris* | 2004 | Mexico | GU906909 |
| GC 30 | *L. stylirostris* | 2004 | Mexico | GU906902 |
| GC 31 | *L. stylirostris* | 2004 | Mexico | GU906910 |
| GC 37 | *L. stylirostris* | 2004 | Mexico | GU906911 |
| GC 41 | *L. stylirostris* | 2004 | Mexico | GU906916 |
| GC 42 | *L. stylirostris* | 2004 | Mexico | GU906912 |
| GC 44 | *L. stylirostris* | 2004 | Mexico | GU906913 |
| GC 45 | *L. stylirostris* | 2004 | Mexico | GU906903 |
| GC 47 | *L. stylirostris* | 2004 | Mexico | GU906914 |
| GC 51 | *L. stylirostris* | 2004 | Mexico | GU906915 |
| PP 17 | *L. stylirostris* | 2005 | Mexico | GU906917 |
| PP 18 | *L. stylirostris* | 2005 | Mexico | GU906918 |
| PP 20 | *L. stylirostris* | 2005 | Mexico | GU906919 |
| PP 22 | *L. stylirostris* | 2005 | Mexico | GU906920 |
| PP 24 | *L. stylirostris* | 2005 | Mexico | GU906921 |
| PP 25 | *L. stylirostris* | 2005 | Mexico | GU906922 |
| PP 29 | *L. stylirostris* | 2005 | Mexico | GU906923 |
| PP 30 | *L. stylirostris* | 2005 | Mexico | GU906924 |
| BK 02 | *L. stylirostris* | 2004 | Mexico | GU906929 |
| BK 03 | *L. stylirostris* | 2004 | Mexico | GU906934 |
| BK 05 | *L. stylirostris* | 2004 | Mexico | GU906933 |
| BK 11 | *L. stylirostris* | 2004 | Mexico | GU906930 |
| BK 13 | *L. stylirostris* | 2004 | Mexico | GU906931 |
| **Isolate Name** | **Host Species** | **Isolation Year** | **Geographic Location** | **Accession Number** |
| BK 26 | *L. stylirostris* | 2004 | Mexico | GU906932 |
| BK 27 | *L. stylirostris* | 2004 | Mexico | GU906927 |
| BK 29 | *L. vannamei* | 2004 | Mexico | GU906925 |
| BK 39 | *L. vannamei* | 2004 | Mexico | GU906928 |
| EM 01 | *L. stylirostris* | 2004 | Mexico | GU906935 |
| EM 04 | *L. stylirostris* | 2004 | Mexico | GU906936 |
| EM 05 | *L. stylirostris* | 2004 | Mexico | GU906948 |
| EM 10 | *L. stylirostris* | 2004 | Mexico | GU906937 |
| EM 12 | *L. stylirostris* | 2004 | Mexico | GU906938 |
| EM 16 | *L. stylirostris* | 2004 | Mexico | GU906949 |
| EM 18 | *L. stylirostris* | 2004 | Mexico | GU906939 |
| EM 26 | *L. stylirostris* | 2004 | Mexico | GU906940 |
| EM 27 | *L. stylirostris* | 2004 | Mexico | GU906946 |
| EM 29 | *L. stylirostris* | 2004 | Mexico | GU906947 |
| EM 33 | *L. stylirostris* | 2004 | Mexico | GU906941 |
| EM 34 | *L. stylirostris* | 2004 | Mexico | GU906942 |
| EM 35 | *L. stylirostris* | 2004 | Mexico | GU906943 |
| EM 37 | *L. stylirostris* | 2004 | Mexico | GU906944 |
| EM 38 | *L. stylirostris* | 2004 | Mexico | GU906945 |
| CU 11 | *L. stylirostris* | 2005 | Mexico | GU906957 |
| CU 13 | *L. vannamei* | 2005 | Mexico | GU906950 |
| CU 15 | *L. stylirostris* | 2005 | Mexico | GU906958 |
| CU 19 | *L. stylirostris* | 2005 | Mexico | GU906963 |
| CU 20 | *L. stylirostris* | 2005 | Mexico | GU906960 |
| CU 21 | *L. stylirostris* | 2005 | Mexico | GU906951 |
| CU 23 | *L. stylirostris* | 2005 | Mexico | GU906952 |
| CU 25 | *L. stylirostris* | 2005 | Mexico | GU906953 |
| CU 26 | *L. vannamei* | 2005 | Mexico | GU906954 |
| CU 27 | *L. vannamei* | 2005 | Mexico | GU906955 |
| CU 31 | *L. stylirostris* | 2005 | Mexico | GU906956 |
| CU 32 | *L. stylirostris* | 2005 | Mexico | GU906961 |
| CU 35 | *F. californiensis* | 2005 | Mexico | GU906962 |
| CU 37 | *L. stylirostris* | 2005 | Mexico | GU906959 |
| BM 16 | *L. stylirostris* | 2004 | Mexico | GU906971 |
| BM 17 | *L. stylirostris* | 2004 | Mexico | GU906977 |
| BM 20 | *L. stylirostris* | 2004 | Mexico | GU906972 |
| BM 23 | *L. stylirostris* | 2004 | Mexico | GU906973 |
| BM 26 | *L. stylirostris* | 2004 | Mexico | GU906974 |
| BM 29 | *L. stylirostris* | 2004 | Mexico | GU906975 |
| BM 30 | *L. stylirostris* | 2004 | Mexico | GU906976 |
| BM 01 | *L. stylirostris* | 2004 | Mexico | GU906964 |
| **Isolate Name** | **Host Species** | **Isolation Year** | **Geographic Location** | **Accession Number** |
| BM 03 | *L. stylirostris* | 2004 | Mexico | GU906968 |
| BM 05 | *L. stylirostris* | 2004 | Mexico | GU906969 |
| BM 13 | *L. stylirostris* | 2004 | Mexico | GU906970 |
| BM 15 | *L. stylirostris* | 2004 | Mexico | GU906966 |
| BM 27 | *L. stylirostris* | 2004 | Mexico | GU906967 |
| MxPsAF218266 | *L. stylirostris* | 1986 | Hawaii | AF218266 |
| AuPmEU675312 | *P. monodon* | 1993 | Australia | EU675312 |
| MxPsAF273215 | *L. stylirostris* | 1998 | Mexico | AF273215 |
| ThPmAY102034 | *P. monodon* | 2000 | Thailand | AY102034 |
| TwPmAY355306 | *P. monodon* | 2001 | Taiwan | AY355306 |
| TwPmAY355307 | *P. monodon* | 2001 | Taiwan | AY355307 |
| EAfPmAY124937 | *P. monodon* | 2002 | East Africa | AY124937 |
| EcPvAY362548 | *P. vannamei* | 2003 | Ecuador | AY362548 |
| TwPmAY355308 | *P. monodon* | 2003 | Taiwan | AY355308 |
| TwPmAY362547 | *P. monodon* | 2003 | Taiwan | AY362547 |
| TzPmDQ228358 | *P. monodon* | 2004 | Mexico | DQ228358 |
| ChPmEF633688 | *P. monodon* | 2007 | China | EF633688 |
| InPmFJ169961 | *P. monodon* | 2007 | India | FJ169961 |
| InPmGQ411199 | *P. monodon* | 2007 | India | GQ411199 |
